# Effect of MIND diet intervention on cognitive performance and brain structure in healthy obese women: a randomized controlled trial

**DOI:** 10.1038/s41598-021-04258-9

**Published:** 2022-02-21

**Authors:** Golnaz Arjmand, Mojtaba Abbas-Zadeh, Mohammad Hassan Eftekhari

**Affiliations:** 1 Department of Clinical Nutrition, School of Nutrition and Food Sciences, Shiraz University of Medical Science, 71645-111 Shiraz, Iran; 2 School of Cognitive Sciences, Institute for Research in Fundamental Sciences (IPM), Tehran, Iran

**Keywords:** Neuroscience, Diseases, Endocrinology, Medical research

## Abstract

Previous studies suggested adherence to recently developed Mediterranean-DASH Intervention for Neurodegenerative Delay (MIND) associated with cognitive performance. This study aimed to examine the effect of MIND dietary pattern on cognitive performance features and changes in brain structure in healthy obese women. As a total of 50 obese women were assessed for eligibility, we randomly allocated 40 participants with mean BMI 32 ± 4.31 kg/m^2^ and mean age 48 ± 5.38 years to either calorie-restricted modified MIND diet or a calorie-restricted standard control diet. Change in cognitive performance was the primary outcome measured with a comprehensive neuropsychological test battery. We also performed voxel-based morphometry as a secondary outcome to quantify the differences in brain structure. All of the measurements were administered at baseline and 3 months follow-up. Thirty-seven participants (MIND group = 22 and control group = 15) completed the study. The results found in the MIND diet group working memory + 1.37 (95% CI 0.79, 1.95), verbal recognition memory + 4.85 (95% CI 3.30, 6.40), and attention + 3.75 (95% CI 2.43, 5.07) improved more compared with the control group (*ps* < 0.05). Results of brain MRI consist of an increase in surface area of the inferior frontal gyrus in the MIND diet group. Furthermore, the results showed a decrease in the cerebellum-white matter and cerebellum-cortex in two groups of study. Still, the effect in the MIND group was greater than the control group. The study findings declare for the first time that the MIND diet intervention can reverse the destructive effects of obesity on cognition and brain structure, which could be strengthened by a modest calorie restriction.

**Clinical trial registration** ClinicalTrials.gov ID: NCT04383704 (First registration date: 05/05/2020).

## Introduction

Obesity, characterized by excess accumulation of fat mass, is considered as one of the most growing health issues facing the world^1^. Aside from the known metabolic and physiological concomitant medical risks, research supports the view that higher body mass index (BMI) is associated with alteration in global cognitive performance^2^ as well as overall brain volume^3,4^.

However, there is no conclusive evidence of a link between late-life obesity and dementia^5^. Middle-aged obesity has been consistently recognized in epidemiological studies as a risk factor for dementia and Alzheimer's disease (AD)^6^. Recent research shows that obesity in middle age is not only associated with an increased risk of dementia in old age but also with a decrease in the rate of cognitive function in middle age itself. These findings suggest that obesity may affect cognitive functions in the middle-life before any age-related dementia occurs^7^*.* Furthermore, the results of a review study concluded that obesity in middle age is associated with cognitive deficits in the areas of language, motor, and memory function, with deficiencies that are consistently evident in the field of executive function^3^. A longitudinal cross-sectional study in over 2000 middle-aged adults revealed that overweight and obese people recall fewer words and took a long time to complete the cognitive tests compared to normal-weight participants^8^. Similar results observed in a wide age range (20–82 years) research on obesity and overweight individuals illustrated that obesity without interaction with aging also has a devastating effect on cognitive performance^7^. It is widely known the hippocampal formation, which plays a crucial role in learning and memory^9^, is particularly impacted by obesity and aging^10^, and its small size predicts cognitive impairment and dementia in individuals^11,12^. according to neurological investigations, hippocampal atrophy due to obesity in middle age has been associated with an increased risk of cognitive deterioration in the elderly^13,14^.

As reported in a population-based cohort study of healthy adults which used magnetic resonance image-based brain anatomy, in 527 individuals aged 20–87 years, a greater degree of atrophy in cerebral white matter volume in overweight and obese participants is associated with maximal effects in middle age^15^. These findings imply that obesity in middle age is a powerful predictor of dementia in the elderly. Being obese in middle age raises the likelihood of functional impairment and brain disease^16^. Therefore, if obesity in mid-life threatens cognition, obesity intervention (e.g., weight loss) may reduce the risk of dementia in the future.

In addition, a negative relationship between BMI and global cognitive performance, even when controlled for cognitive aging, was observed in the previous studies^7^. According to the study, those who are obese (BMI > 30 kg/m^2^) or overweight (BMI = 25–29.9 kg/m^2^) in middle age had a higher risk of dementia and Alzheimer's disease than people who are not and the relative risk is 2.04 and 1.64 respectively^17^.

The researchers discovered that obese people (mean BMI 39 kg/m^2^) had lower gray matter densities in the post-center gyrus, frontal lobe, putamen, and forehead than people with a BMI of 22 kg/m^2^^18^. Another study looked at whether bariatric surgery reduced the occurrence of moderate cognitive impairment (MCI) in 171 persons (mean age = 43.07 ± 1.21). MCI was shown to be widespread in young and middle-aged persons with severe obesity, and the prevalence of MCI decreased after obesity surgery, according to the findings^19^. So, based on the above studies, the occurrence of obvious cognitive disorders such as MCI is unavoidable in people with a BMI greater than 35 kg/m^2^.

However, it is not precisely clear how obesity can affect cognitive performance and brain structure. It has been found that negative consequences of obesity itself, before the occurrence of the related diseases, can lead to cognitive impairments^4^. These deficits were observed by using a 5-day high-fat diet (75% energy) in healthy young men, showing that this duration was sufficient to reduce the speed of retrieval and attention^20^. Specifically, excessive energy intake and obesity go with chronically elevated pro-inflammatory factors such as amyloid-beta and homocysteine, which can impair neurons' ability to adapt to chronic inflammation^21^ and oxidative stress^22^. Brain-Derived Neurotrophic Factor (BDNF) is another crucial regulator component that is widely recognized in the development, survival, and differentiation of the nervous system and is functionally applied in memory and cognitive abilities^23^. Evidence from animal studies showed that BDNF stimulates neurogenesis, which may contribute to the beneficial effects of BDNF on cognitive function^24^. In a survey conducted on Sprague–Dawley rats, it has been shown that a 2-month high-fat diet disrupts their cognitive function, and these behavioral disorders have been associated with a decrease in BDNF levels and synaptic function markers^25^. Therefore, assessment of these factors can play a role in measuring the effect of dietary patterns on cognitive performances in obese people.

Although, only a handful of experimentally controlled studies measured the effect of diet on cognition and brain volume. Observations in human studies provided emerging insight into modifiable lifestyle factors, such as dietary patterns^26^. In particular, evidence from epidemiologic studies suggested that the Mediterranean and Dietary Approach to Stop Hypertension (DASH) diet can have protective effects on cognitive decline, but results are inconsistent^27,28^. A prospective population-based study that included 3831 men and women > 65 years old revealed that higher DASH and Mediterranean diet scores are associated with a higher average of Modified Mini-Mental State Examination Survey test (MMSE)^29^. Nonetheless, neither of these two dietary patterns have been explicitly raised designed for brain health.

In 2015, Morris and colleagues developed a new brain-protection pattern. This diet has been designed after the Mediterranean and DASH diet to improve some of their dietary factors and have the highest impact on brain health and cognitive performance^30^. The MIND diet emphasizes fruits, mainly berries, green leafy vegetables, nuts, olive oil, whole grains, fish, beans, and poultry. The MIND diet also limits the consumption of butter, cheese, red meat, fried foods, and sweets. To investigate whether the MIND diet can slow cognitive decline with aging, Morris et al. examined 960 participants over an average of 4.7 years to show that the MIND diet slows the process of reducing age-related cognitive abilities, including episodic memory, semantic memory, and the speed of perception of concepts.

Additionally, a higher score for the MIND dietary pattern is associated with a reduction in the rate of cognitive dysfunction in healthy older adults^30,31^. It seems that increased sensitivity to long-term effects of oxidative stress and inflammation due to obesity on the nervous system can reduce the cognitive and motor function of the brain. Therefore, a MIND diet with high levels containing polyphenols and antioxidant components can reverse the mechanism of oxidative stress and inflammation.

It is predicted that obesity in middle age is not only associated with an increased risk of dementia in later life but also with midlife deficits in domains of language, motor function, memory performance, and most consistently implicated in executive function. These findings suggest that obesity may affect cognition before any cognitive decline appears. Therefore, if obesity can destroy cognition in midlife, obesity-related intervention may improve this defective cycle^32^. To the best of our knowledge, the majority of the previous MIND diet studies have been cross-sectional and longitudinal, focusing on elderly participants. Here, we designed a randomized controlled trial to address this question of whether MIND dietary pattern can improve cognitive performance in middle-aged obese individuals. For this purpose, we assessed the changes in cognitive performance and the brain structures in healthy obese women.

## Material and methods

### Participants and sample size

This current randomized, well-controlled trial was conducted on 37 randomly selected participants signed up through public advertising at Imam Reza clinic of Shiraz University of Medical Sciences. To calculate sample size, we use the formula suggested for randomized clinical trials. We considered the type I error of 5% (α = 0.05), type II error of 20% (β = 0.02, power = 80%), and working memory capacity as a primary outcome variable^33^. Based on McMillan's paper (2011), we utilized cognitive performance as the primary measure to calculate sample size, and we ended up with 11 participants in each group^34^. We expanded the sample size to account for an estimated drop-out rate.

#### Inclusion and exclusion criteria

The inclusion criteria were defined as middle-aged women (40–60 years), without any metabolic complication including diabetes, thyroid diseases, anemia, lipid disorders, and cardiovascular diseases, BMI 30–35 kg/m^2^, MMSE ≥ 24, and no history of severe untreated medical, neurological, and psychiatric illnesses which include depression, Parkinson's disease, Alzheimer disease, psychiatric conditions, mental disorders, the onset of stroke or transient ischemic attack within the previous 3 months; history of brain injury, liver disease, Hepatitis C, or HIV; illnesses and conditions that are associated with weight change; and diagnosis of cancer within the previous five years that may interfere with the study intervention.

At first, the main aim of our study was to investigate the relationship between the MIND diet and cognitive performance in obese adults (both men and women). But at the beginning of the study, we noticed that the male population had low compliance with the studied instructions. Therefore, in the main study, only the group of women was included*.*

We also included participants who did not have gastrointestinal problems, did not participate in weight loss programs or did not use weight loss drugs in the last 3 months. In this present study, we excluded those participants who had not wholly followed the dietary pattern or became pregnant and underwent special medical treatments during 3 months follow up. The eligibility of participants was first asked during a telephone screening.

### Procedure

Participants were randomly allocated to the calorie-restricted control diet and calorie-restricted modified MIND diet group using a computerized, web-based random number table. The same dietitian who was blinded generated the random allocation sequence and assigned participants to each group. At the initial visit, participants underwent a face-to-face standardized medical interview and also a neurological examination. Demographic and anthropometric data, as well as details on dietary intakes, have been gathered. Nutritional data were collected using a 168-item semi-quantitative food frequency questionnaire (FFQ) to know the usual dietary intake of participants^35^. Before and after the 3 months of study, participants underwent a comprehensive neuropsychological test as well as structural neuroimaging.

Since our intervention is a dietary intervention, it was not possible to blind the study groups. However, these groups did not have any contact with each other. And since our control group was active and received a calorie-restricted diet, there is no emphasis on the superiority of any diet in either group. To avoid bias in the study, the individuals who collected the study output were separated from those who delivered the intervention. Classification of individuals in the study groups was done in a separate center that had no direct contact with the participants.

All study protocols were approved by the Ethics Committee of Shiraz University of Medical Science, and participants were explained the ethical aspect of the study. Participants also provided signed informed consent before participation following the Declaration of Helsinki Law (IR.SUMS.REC.1397.759). The full date of the first registration and the registration number is 05/05/2020 and ClinicalTrials.gov ID: NCT04383704, respectively.

### Diets

The MIND dietary pattern used for this study was based on the MIND diet developed by Morris and colleagues in 2015^30^. Participants in the MIND diet group received instruction in modifying the content of their diet to meet MIND pattern guidelines. This pattern emphasizes natural, plant-based foods, promoting increased consumption of berries and green leafy vegetables, whole grain cereals, fish, nuts, and olive oil, with limited intake of animal-based and high saturated fat foods. The component and scoring of the MIND diet are shown in Table 1. In the current study, wine consumption was not included because its usage is forbidden in our country. Therefore, we encourage our participants to use grape and grape juice, as well as raisins and currant, to modify servings.

**Table 1:** MIND diet components and scoring.

MIND diet components	Score		
	0	0.5	1
Green leafy vegetables^α^	≤ 2 serving/week	> 2 to < 6/week	≥ 6 serving/week
Other vegetables^β^	≤ 5 serving/week	5 to < 7/week	≥ 1 serving/day
Berries	≤ 1 serving/week	1 to 2/week	≥ 2servings/week
Nuts	< 1/month	1/month to < 5/week	≥ 5 servings/week
Olive oil	Not primary oil	–	primary oil
Butter, margarine	> 2 table spoon/day	1 to 2 table spoon/day	< 1 table spoon/day
Cheese	≥ 7 servings/week	≥ 1 to < 7/week	< 1 servings/week
Whole grains	< 1 serving/day	≥ 1 to < 3/day	≥ 3 servings/day
Fish (not fried)	Rarely	1 to 3/month	≥ 1 meals/week
Beans^€^	< 1 meal/week	1 to 3/week	> 3meal/week
Poultry (not fried)	< 1 meal/week	≥ 1 to < 2/week	≥ 2 meals/week
Red meat and products	> 6 meals/week	≥ 4 to ≤ 6/week	< 4 meals/week
Fast fried foods	≥ 4 times/week	1 to < 4/week	< 1 time/week
Pastries and sweets	≥ 7 servings/week	≥ 5 to < 7/week	< 5 servings/week
Wine	> 1 glass/day or never	1/month to 6/week	1 glass/day
Total score	0	7.5	15

^α^Kale, collards, greens; spinach; lettuce/tossed salad.

^β^Green/red peppers, squash, cooked carrots, raw carrots, broccoli, celery, potatoes, peas or lima beans, tomatoes, tomato sauce, string beans, beets, corn, zucchini/summer squash/eggplant, coleslaw, potato salad.

^€^Beans, lentils, soybeans.

A 7-day menu was then developed, meeting the required number of servings per day every week. The participants did not receive any financial compensation or gifts, but participants had to adopt the recommended diet strictly by themselves after intensive instruction and education. Participants were followed up by the same dietitian every week. Dietary intervention adherence was measured via assessment, (1) MIND diet score questionnaire, and 3-day food recall. Each recorded meal and snack were rated as either following the MIND diet or not. Participants were classified as having successfully adhered to the MIND diet if 80% or more of their meals/snacks met this criterion. Daily calorie intake was individually specified in both study groups based on the World Health Organization (WHO) formula for overweight and obese women > 19 years and older^36^. Our goal of reduced calories was to observe the ethical principles and increase individuals' adherence to the two study groups. Therefore, a maximum reduction of 500 cal according to weight and pre-calorie intake has been used as a basis. Meals in both calorie-restricted diet groups included a balanced mix of foods with 50–55% carbohydrates, 30% fat, and 15–20% proteins. We used Nutritionist IV software (First Databank, San Bruno, CA, USA version 3.5.2.) modified for Iranian food to assess the nutritional value of the participants' diet.

Participants in the control group were instructed to calorie-restricted diet alone during the period of study. The control group also received general oral and written information about healthy food choices due to ethical concerns and to keep participants in the study. All parameters were collected at baseline and the end of 3 months of intervention.

### Primary endpoint

#### Assessment of cognitive performance

All of the participants were tested on a complex battery of neurocognitive tests to examine their cognitive performance in different domains. An assistant who was thoroughly trained in the method of performing and measuring cognitive batteries and was completely blind to the study groups assessed cognitive function.

Verbal short memory composite included the score from Forward Digit Span Task (FDST) and Backward Digit Span Task (BDST). The working memory capacity was measured by the Letter Number Sequencing Task (LNST). Attention and visual scanning were obtained by the Symbol Digit Modality Task (SDMT). Auditory Verbal Learning Test (AVLT) was used to test verbal recognition memory performance^37^. Finally, the Trail making test A and B were performed to measure executive function and task switching. We also assessed the ability to inhibit cognitive interference by a well-known Stroop task.

### Secondary endpoint

#### Clinical and anthropometric data

Body weight was measured using a digital scale nearest 0.1 kg, and standing height was measured using a stadiometer to the nearest 0.1 cm. Body composition was obtained with a Bioelectrical Impedance Analysis (BIA) device according to a standardized protocol (In Body S-10, USA). Participants were instructed not to participate in extreme physical activity or consume alcohol and caffeinated beverages within 24 h before the measurement.

After overnight fasting, blood samples were collected from all participants to determine plasma levels of Brain-derived neurotrophic factor (BDNF) as well as Amyloid-beta and homocysteine by ELISA test using the specialized kit before and after 3 months follow up.

Initially, before starting the study, all participants were asked to report their level of physical activity using the International Physical Activity Questionnaires (IPAQ) short form. At the beginning and throughout the study, it was explained that they do not allow to change their level of physical activity. Every month this questionnaire is filled out again for the participants. In face-to-face meetings and telephone calls, adherence to the study protocols is emphasized again.

#### Image acquisition and processing

Due to the high cost of magnetic resonance imaging as well as the lack of accompaniment of participants because of fear of being in the device, it was decided that only a subset of participants, who are the initial number of people determined by sample size calculation, be placed under examination with MRI.

Magnetic resonance imaging was carried out by a 3 T Siemens Skyra system with a 12-channel head coil on a subgroup of 11 participants in each group. Pre- and post-scans involved high-resolution T1-weighted anatomical images. T1-weighted images consisted of 192 slices and were used in the sagittal plane, prepared with gradient-echo sequence (repetition time = 1900 ms, echo time = 2.52 ms, flip angle = 9°, voxel size = 1 × 1 × mm). Image preprocessing was performed by the Freesurfer, stable version 5.1 (http://surfer.nmr.mgh.harvard.edu).

To capture the changes in brain structure in response to MIND diet intervention in study groups and reduce biased analysis and measurement noise, the longitudinal processing procedure of Freesurfer was used. For this procedure, first, the images from baseline and 3 months were independently processed. Then, the baseline data was subtracted from the 3 months data in a vertex-wise manner. For group analysis, the output map for each participant was resampled, normalized to common space, and smoothed with a Full Width of Half Maximum (FWHM) of 10 mm. Smoothing was only performed for vertex-wise whole-brain analyses, and it was not used for parcellation measures of cortical regions and volumetric measurements of subcortical regions.

### Statistical analysis

Data were analyzed using SPSS 22.0 for Windows (SPSS, Chicago, IL), and the level of significance for all analyses was adjusted at alpha *p* < 0.05. To screen the normality of variables, a one-sample Kolmogorov-Smirnow test was used. Descriptive statistics for neuropsychological data and physical parameters are performed as mean ± SEM. Independent sample t-tests were used to determine the differences between baseline measures of two groups. A paired sample t-test was used to compare the data at the baseline and the 3 months of intervention for each group. We also used the Man-Whitney as a non-parametric test to compare the differences in brain structure between the two groups. A mixed model two-way repeated-measure analysis of variance (ANOVA) was performed to compare the mean differences between groups that had been divided into two factors, where time (baseline, 3 months) as a within-participant factor and treatment (MIND diet group, control group) as a between-participant factor.

The effect of diet intervention in the whole brain and Regions of Interest (ROIs) was statistically tested. For whole-brain analysis, output maps were corrected for multiple comparisons using the false discovery rate (FDR) 0.05 as implemented in Freesurfer. Corresponding effect sizes in partial Eta square were calculated to evaluate the magnitude of effect for all hypotheses.

### Ethics approval

We ensure that this work has been carried out in accordance with the Code of Ethics of the World Medical Association (Declaration of Helsinki) for experiments involving humans, and informed consent was obtained from all subjects. The study protocol was approved by the institutional ethics board of the Shiraz University of Medical Sciences.

## Results

### Participant characteristics

**Figure 1:**
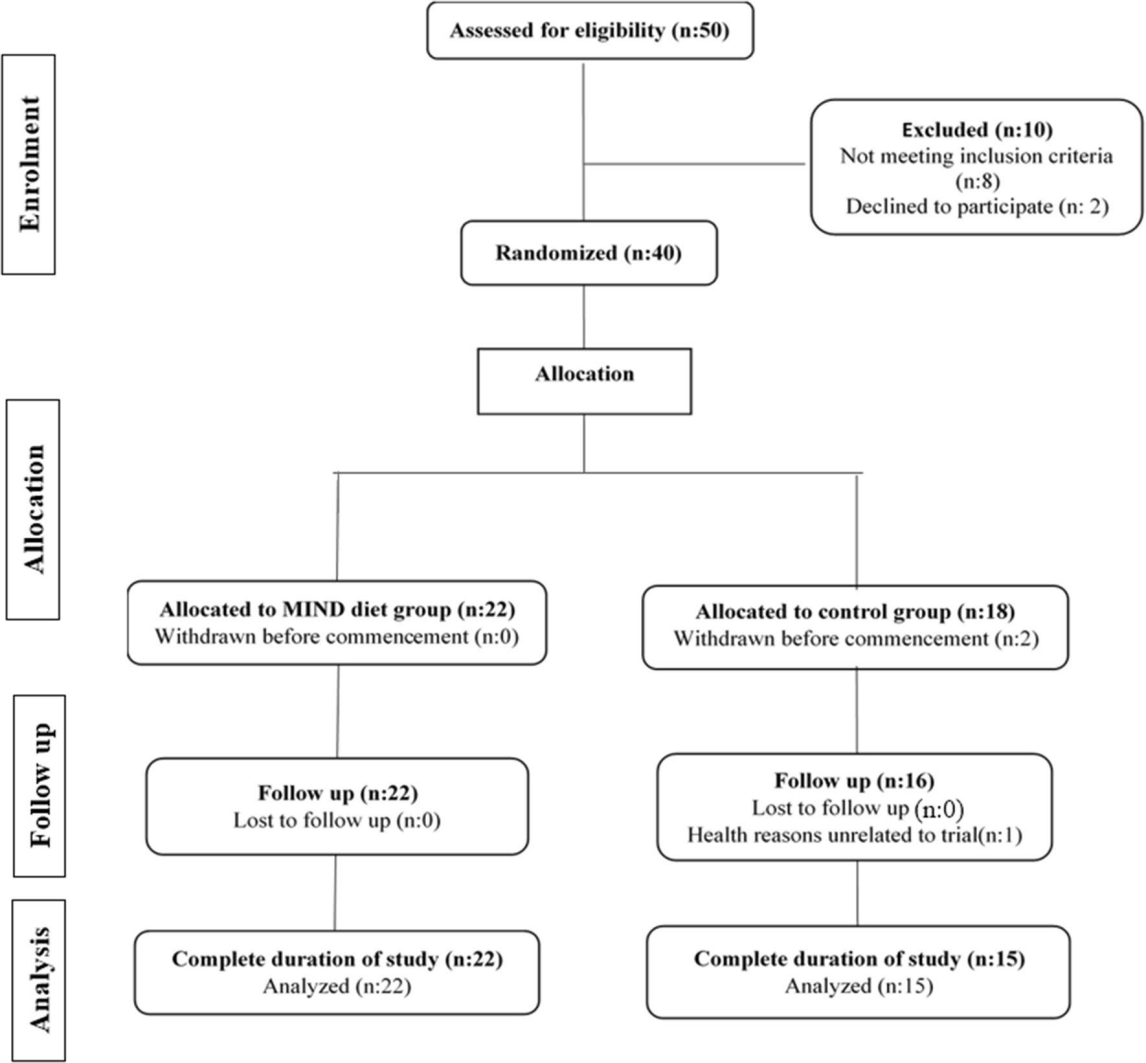
CONSORT flow diagram of the study.

**Figure 2:**
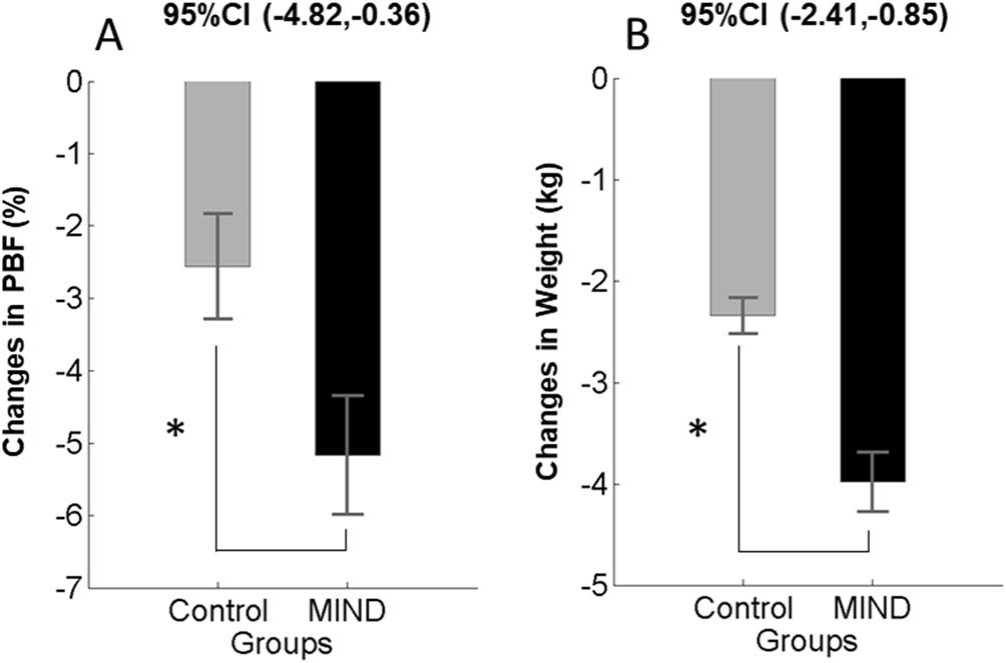
Anthropometric changes (mean and standard error of the mean) in the MIND diet group (black color) and control group (gray color) at baseline and follow-up. Note that *p *value < 0.05 in a repeated measure ANOVA test indicates a significant improvement in weight (**A**) and percent of body fat (**B**) in the MIND diet group in comparison with the control group. *Abbreviation* PBF, percent of body.

The study took place between October 2018 and March 2019. We screened 50 volunteers for inclusion criteria, and 40 people met the inclusion criteria. The analyzed study consisted of 37 participants (n = 22 MIND diet group, n = 15 control group). The CONSORT flowchart diagram has been reported in Fig. 1. The participants in both groups completed the study, and no side effects have been reported. Descriptive results showed that at baseline, participants' mean (SD) age was 48 ± 5.3 years, and the majority of them were married (83.8%). All of the participants who participated in the study were right-handed^38^. Table 2 shows the baseline characteristics of the participants who participated in the study. The MIND diet and control groups had similar clinical characteristics, and no participants reported a history of diabetes and hypertension. To examine anthropometric parameters over 3 months, a repeated measure ANOVA analysis using the variables as within-subject factor and the type of treatments as a between-subject factor was performed. Figure 2 shows that significant group × time interaction for the body weight and percent of body fat after a 3-month intervention in the MIND diet group compared with the control group (*ps* < 0.05, Fig. 2A, B).

**Table 2:** Baseline characteristics of participants according to the group studies.

Variables	MIND diet (n:22)	Control group (n:15)	*p *value
Age (years)	48.95 (1.07)*	48.86 (1.56)	0.962
Education (years)	16.40 (0.22)	16.40 (0.27)	0.980
Weight (kg)	81.95 (2.33)	82.33 (3.96)	0.930
Height (cm)	160.18 (0.99)	159.60 (1.42)	0.731
Percent of body fat (%)	40.84 (1.13)	41.05 (1.63)	0.913
Fat Free Mass (kg)	48.05 (1.09)	47.66 (1.37)	0.823
BMI (kg/m^2^)	31.90 (0.79)	32.19 (1.28)	0.839
WC (cm)	99.75 (2.08)	103.69 (3.25)	0.292
MMSE (score)	26.22 (0.34)	26.73 (0.45)	0.375
IPAQ score (MET/min/day)	512.01 (26.13)	567.40 (25.19)	0.416

*Mean (SEM) = Baseline differences between groups used t-test analysis.

BMI, Body Mass Index; WC, Waist circumference; MMSE, Mini-Mental State Examination survey; MET, Metabolic Equivalent; IPAQ, International Physical Activity Questionnaires.

### Changes in food intake

Table 3 showed no significant difference between the two groups in terms of energy and nutrient intake at the beginning of the study. After a 3-month study follow-up, the amount of fiber intake is significantly higher. The percent of calories received from fats is lower in the MIND diet group than in the control group. Between-group analysis revealed that the amount of all nutrient intake was changed significantly from baseline to the 3 months follow-up.

**Table 3:** Changes in food nutrients from baseline to the final assessment in the MIND diet group vs. control group.

Variables	MIND group	Control group	*p* value^2^
**Energy (Kcal)**			
Baseline	2236 ± 40.02	2233 ± 49.29	0.968
Follow up	1781 ± 33.68▼	1831 ± 45.16▼	0.373
*p* value^1^	< 0.001	< 0.001	
**Energy from protein (%)**			
Baseline	17.72 ± 0.15	18.06 ± 0.13	0.176
Follow up	20.16 ± 0.03▲	20.19 ± 0.16▲	0.849
*p* value	< 0.001	< 0.001	
**Energy from carbohydrate (%)**			
Baseline	45.73 ± 0.65	46.92 ± 1.07	0.323
Follow up	43.91 ± 0.71▼	43.70 ± 0.82▼	0.855
*p* value	0.005	0.007	
**Energy from fat (%)**			
Baseline	35.74 ± 0.80	36.20 ± 0.83	0.705
Follow up	29.83 ± 0.42▼	28.33 ± 0.45▼	0.025
*p* value	< 0.001	< 0.001	
**Saturated fatty acid (%)**			
Baseline	15.12 ± 0.27	15.55 ± 0.43	0.383
Follow up	8.59 ± 0.11▼	8.91 ± 0.24▼	0.861
*p* value	< 0.001	< 0.001	
**Monounsaturated fatty acid (%)**			
Baseline	8.93 ± 0.13	8.52 ± 0.12	0.042
Follow up	12.50 ± 0.20▲	11.30 ± 0.26▲	0.001
*p* value	< 0.001	< 0.001	
**Energy from w-3 (%)**			
Baseline	0.43 ± 0.014	0.47 ± 0.022	0.148
Follow up	0.83 ± 0.035▲	0.54 ± 0.038▲	< 0.001
*p* value	< 0.001	0.182	
**Energy from w-6 (%)**			
Baseline	3.69 ± 0.25	3.67 ± 0.41	0.959
Follow up	5.86 ± 0.37▲	4.47 ± 0.25▲	0.009
*p* value	< 0.001	0.082	
**Fiber (g)**			
Baseline	23.83 ± 0.36	23.18 ± 0.38	0.237
Follow up	41.41 ± 0.84▲	28.53 ± 0.63▲	< 0.001
*p* value	< 0.001	< 0.001	
**Sugar (g)**			
Baseline	97.25 ± 2.54	95.87 ± 2.66	0.717
Follow up	52.81 ± 1.83▼	56.89 ± 2.36▼	0.176
*p* value	< 0.001	< 0.001	

Changes in food nutrient from baseline to the final assessment using independent sample t-test and paired sample t-test.^1^*p* value within groups using paired t-test. ^2^*p* value between groups using independent sample t-test.

▲Increase in nutrient intake at the final assessment compared with baseline. ▼Decrease in nutrient intake at the final assessment compared with baseline.

As shown in Table 4, the consumption of green leafy vegetables, other vegetables, berries, olive oil, fish, whole grains, beans, and poultry significantly increased in participants in the MIND diet group (*p*s < 0.05). The MIND diet group also considerably decreased consumption of butter, cheese, red meats, fast foods, and sweets (*p*s < 0.05). There were no significant changes in nut consumption in the MIND diet group before and after 3 months. Additionally, in the control group, the consumption of green leafy vegetables has increased significantly. On the other hand, the consumption of sweets, butter, and fast foods has also shown a significant decrease in this group.

**Table 4:** Changes in food intake from baseline to follow-up in the MIND diet group vs. control group.

Foods	MIND diet group (n:22)			Control group (n:15)	
			*p* value		*p* value
Green leafy vegetables (serving/week)^α^	Baseline Follow up	3.72 ± 0.55 5.50 ± 0.51▲	< 0.001	3.60 ± 0.50 3.93 ± 0.25▲	< 0.019
Other vegetables (serving/week) ^β^	Baseline Follow up	3.86 ± 0.63 6.90 ± 0.52▲	< 0.001	3.93 ± 0.25 4.00 ± 0.65▲	< 0.670
Berries (serving/week)	Baseline Follow up	0.90 ± 0.42 2.00 ± 0.00▲	< 0.001	0.93 ± 0.25 0.73 ± 0.59▼	< 0.189
Nuts (serving/month)	Baseline Follow up	4.45 ± 1.01 4.63 ± 0.49▲	0.478	4.53 ± 0.63 3.73 ± 0.59▼	< 0.001
Olive oil (primary oil)*	Baseline Follow up	0 1	< 0.001	0 0	1
Butter, margarine (Table spoon/day)	Baseline Follow up	1.50 ± 0.59 0.63 ± 0.49▼	< 0.001	1.20 ± 0.41 0.46 ± 0.51▼	< 0.001
Cheese (servings/week)	Baseline Follow up	6.40 ± 0.50 1.68 ± 0.56▼	< 0.001	6.33 ± 0.48 6.33 ± 0.48	1
Whole grains (serving/day)	Baseline Follow up	1.00 ± 0.00 2.18 ± 0.39▲	< 0.001	1.00 ± 0.00 0.33 ± 0.48▼	< 0/001
Fish (not fried) (meals/month)	Baseline Follow up	1.22 ± 0.42 2.45 ± 0.50▲	< 0.001	1.33 ± 0.48 1.00 ± 0.00▼	< 0.019
Beans (meal/week) ^€^	Baseline Follow up	1.36 ± 0.49 2.92 ± 0.52▲	< 0.001	1.46 ± 0.51 1.60 ± 0.50▲	< 0.164
Poultry (not fried) (meal/week)	Baseline Follow up	2.09 ± 0.29 2.86 ± 0.35▲	< 0.001	2.13 ± 0.35 2.00 ± 0.37▼	< 0.164
Red meat and products (meals/week)	Baseline Follow up	2.36 ± 0.49 2.00 ± 0.00▼	0.002	2.46 ± 0.51 2.40 ± 0.50▼	0.334
Fast fried foods (times/week)	Baseline Follow up	1.18 ± 0.39 0.45 ± 0.50▼	< 0.001	1.26 ± 0.45 0.93 ± 0.23▼	0.019
Pastries and sweets (serving/week)	Baseline Follow up	4.77 ± 0.68 2.50 ± 0.51▼	< 0.001	4.73 ± 0.79 2.60 ± 0.50▼	< 0.001

▲Increase in food intake at the final assessment compared with baseline. ▼Decrease in food intake at the final assessment compared with baseline.

^α^Kale, collards, greens; spinach; lettuce/tossed salad. ^β^Green/red peppers, squash, cooked carrots, raw carrots, broccoli, celery, potatoes, peas or lima beans, tomatoes, tomato sauce, string beans, beets, corn, zucchini/summer squash/eggplant, coleslaw, potato salad. ^€^Beans, lentils, soybeans.

Calculated using MIND diet score questioner and 3-day food recall, completed four times using pair sample *t*-test and *using Man-Whitney *t*-test.

### Changes in cognitive performance

**Figure 3:**
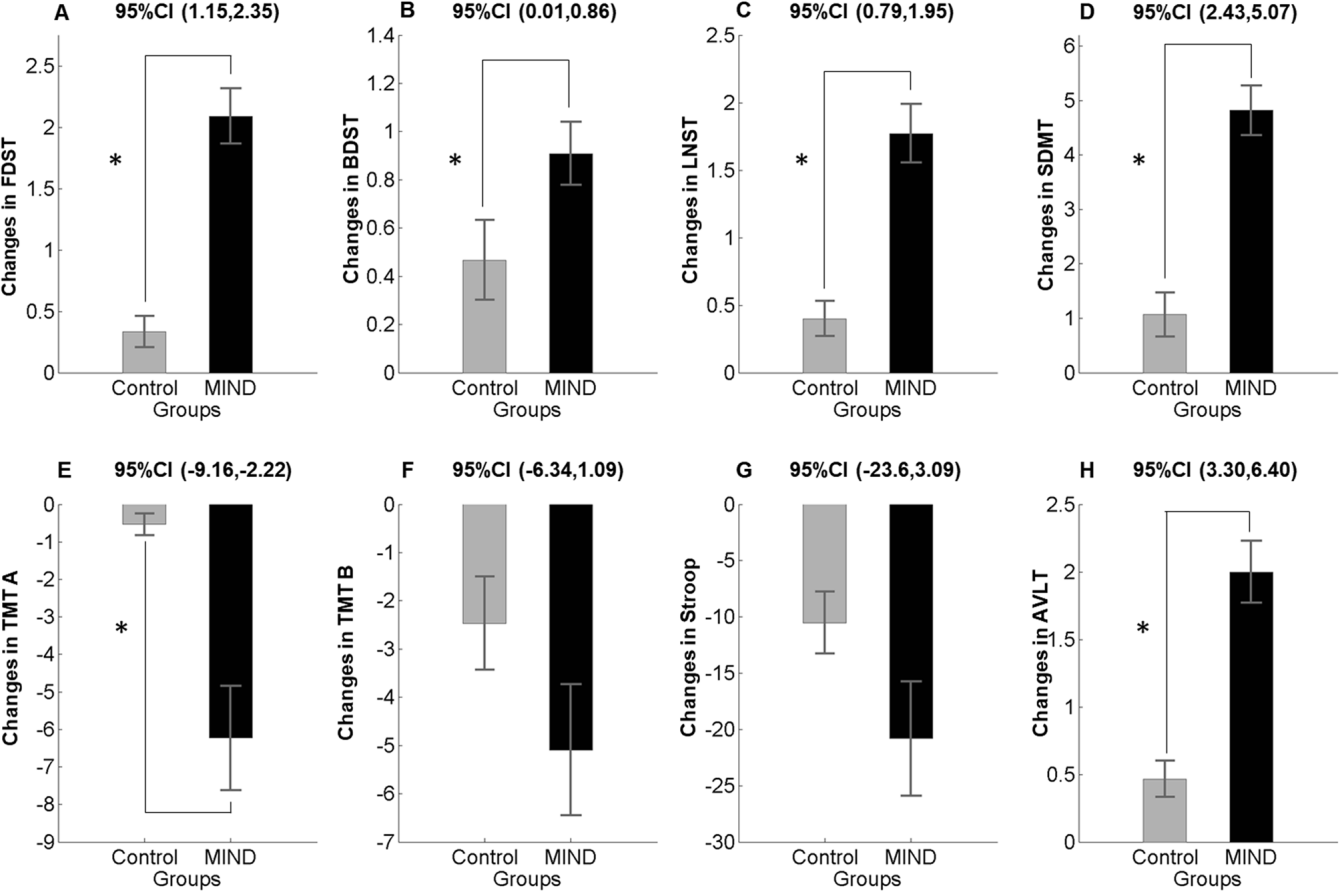
Changes in cognitive performance score (mean and standard error of the mean) in the MIND diet group (black color) and control group (gray color) at baseline and follow-up. *p* value < 0.05 in a repeated measure ANOVA determined that MIND diet intervention significantly altered the mean score of FDST, BDST, LNST, SDMT, TMTA (**A**–**E**) and AVLT (**H**). Similar but not significant trends were found for TMTB and Stroop task (**F**, **G**). *Abbreviation* MIND, Mediterranean-DASH Intervention for Neurodegenerative Delay; FDST, forward digit span task; BDST, Backward Digit Span Task, LNST, letter Number Sequencing Task; SDMT, Symbol Digit Modalities Task; TMTA, Trail Making Test A; TMTB, Trail Making Test B; AVLT, Auditory Verbal Learning Test.

By using linear mixed model analysis, we found that there was a statistically significant effect of time on MIND diet group induced cognitive test score of FDST, BDST, LNST, and SDMT (*p*s < 0.05), indicated the rate of these changes over time was not similar in both groups (Table 5). However, no significant group × time interaction could be found in Trail making test B (*p* = 0.161 Fig. 3F) and Stroop test (*p* = 0.128 Fig. 3G) but, the 3 months intervention had a statistically significant effect on Trial making test A (*p* = 0.002 Fig. 3E). A repeated measure ANOVA with a Greenhouse–Geisser correction determined that the mean Auditory verbal learning task differed significantly between two-time points (*p* = 0 < 0.001 Fig. 3H). The within-group comparison revealed that improvements in all cognitive tests were detected in both the MIND and the control group, which could be part of the results of the learning effect and being familiar with the content of tests (Table 5). Differences in each group's cognitive performance tests at each time point were presented in Fig. 3.

**Table 5:** Cognitive performance data outcomes from group × time interaction after a three-month follow-up study in the MIND diet group versus the control group.

Outcome cognitive performance	Mean (SD)		F (1,35)^α^	*p* value for repeated measure ANOVA^β^			Effect size (Partial eta squared)^€^		
	MIND (n:22)	Control (n:15)	Interaction term	Between group	Within group	Interaction term	Between group	Within group	Interaction term
**FDST**									
Baseline	7/09 (0.41)	6.86 (0.41)	35.515	0.039	≤ 0.001	**≤ 0.001**	0.116	0.659	0.504
3 months	9.18 (0.29)	7.20 (0.35)							
**BDST**									
Baseline	4.90 (0.31)	4.26 (0.30)	4.509	0.034	≤ 0.001	**0.041**	0.122	0.555	0.114
3 months	5.81 (0.25)	4.73 (0.20)							
**LNST**									
Baseline	6.90 (0.43)	5.00 (0.36)	23.101	≤ 0.001	≤ 0.001	**≤ 0.001**	0.421	0.623	0.398
3 months	8.61 (0.29)	5.40 (0.32)							
**SDMT**									
Baseline	42.68 (1.65)	36.26 (2.85)	33.231	0.008	≤ 0.001	**≤ 0.001**	0.184	0.700	0.487
3 months	47.50 (1.52)	37.33 (2.64)							
**TMTA**									
Baseline	39.90 (2.58)	37.00 (2.47)	11.070	0.984	≤ 0.001	**0.002**	0.000	0.309	0.240
3 months	33.68 (1.57)	36.46 (2.40)							
**TMTB**									
Baseline	88.50 (6.47)	101.53 (12.88)	2.048	0.256	≤ 0.001	0.161	0.035	0.327	0.055
3 months	83.40 (5.87)	99.06 (12.00)							
**AVLT**									
Baseline	5.81 (0.43)	5.40 (0.52)	10.485	0.067	≤ 0.001	**≤ 0.001**	0.092	0.662	0.431
3 months	7.81 (0.38)	5.86 (0.45)							
**Stroop**									
Baseline	113.04 (9.37)	150.86 (16.70)	2.434	0.011	≤ 0.001	0.128	0.171	0.393	0.065
3 months	92.27 (6.27)	140.33 (15.34)							

Mixed Model repeated measure ANOVA results of MIND diet group versus control group on cognitive performance after 3 months follow up. Abbreviation: MIND = Mediterranean-DASH Intervention for Neurodegenerative Delay, FDST = forward digit span task, BDST = Backward Digit Span Task, LNST = letter Number Sequencing Task, SDMT = Symbol Digit Modalities Task, TMTA = Trail Making Test A, TMTB = Trail Making Test B, AVLT = Auditory Verbal Learning Test. ^α^F-value (df1 for the numerator, df2 for the denominator), degree of freedom for between-subjects, within-subjects, and the interaction term, ^β^*p* value for mixed repeated measure ANOVA, ^€^Partial eta squared for the ratio of variance associated with an effect. Significant values are in bold.

### Change in neuronal factors

**Figure 4:**
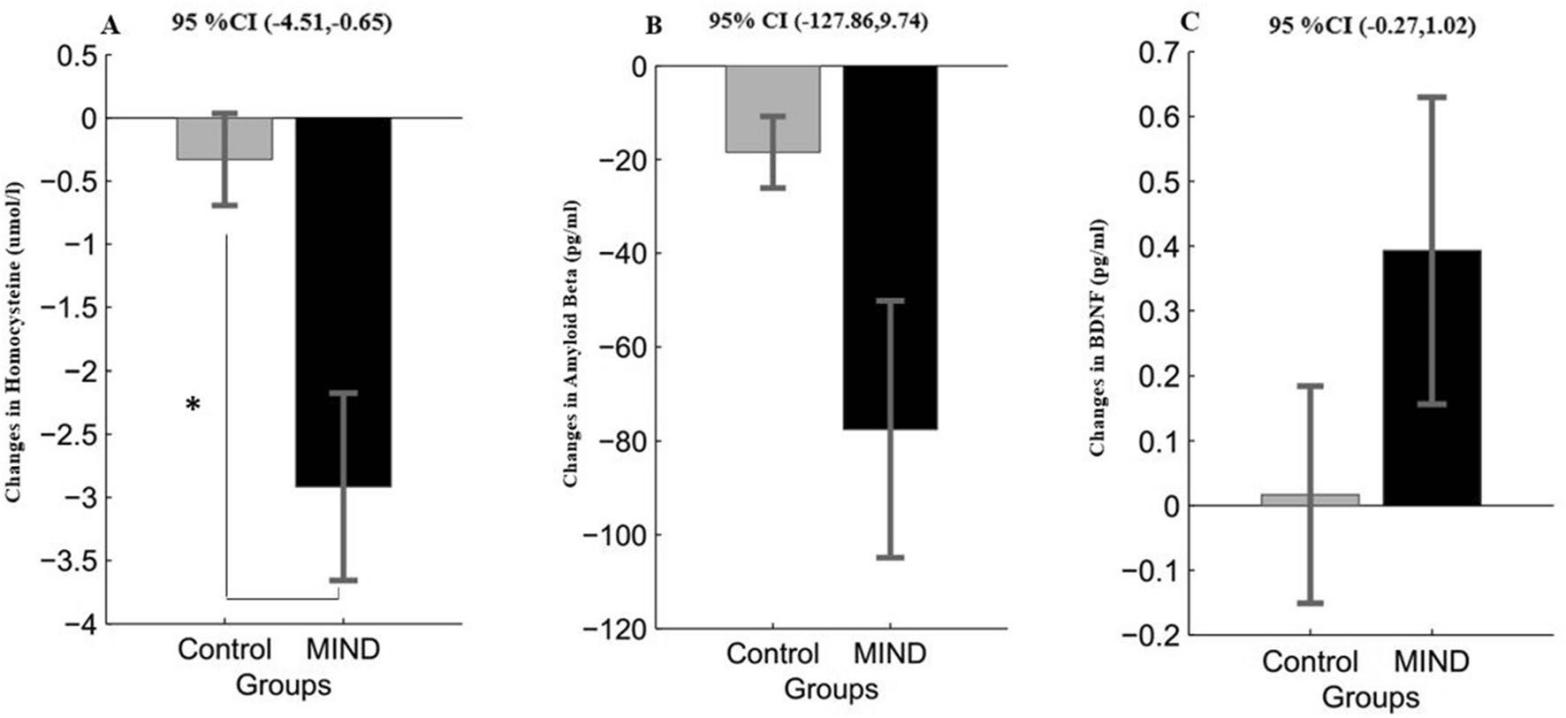
Changes in neuronal factor score (mean and standard error of the mean) in the MIND diet group (black color) and control group (gray color) at baseline and follow-up. *p *value < 0.05 in a repeated measure ANOVA determined that MIND diet intervention significantly altered the mean score of homocysteine (**A**). Similar but not significant trends were found for amyloid beta and BDNF levels (**B**, **C**). *Abbreviation* MIND, Mediterranean-DASH Intervention for Neurodegenerative Delay, BDNF, Brain-derived Neurotrophic factor.

As shown in Fig. 4, the linear mixed repeated measure analysis (ANOVA) for group × interaction revealed, there was a significant interaction effect for plasma levels of homocysteine (*p* = 0.010, Fig. 4A), indicating that homocysteine plasma levels were improved after the 3-month MIND diet interaction. This effect could not be found for the plasma level of Amyloid-beta (*p* = 0.090, Fig. 4B) and BDNF (*p* = 0.248, Fig. 4C). In addition, as a direct indicator of cognitive performance, amyloid-beta levels were more reduced in MIND diet intervention than in the control group. Still, this reduction did not significantly differ between the two study groups (Fig. 3E and Table 4).

### Change in brain structure

**Figure 5:**
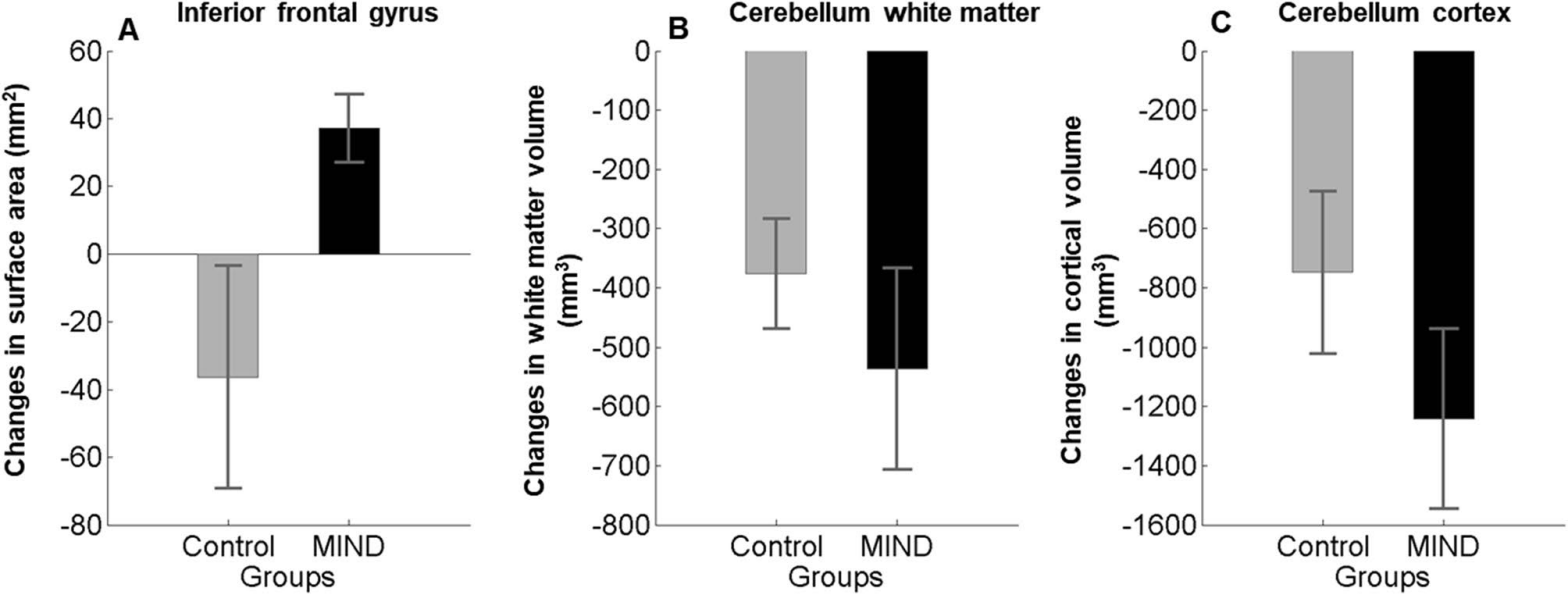
Time × group interaction for gray and white matter volumes of brain regions in the MIND diet group (black color) compared with the control group (gray color). Changes (mean and standard error of the mean) presented. Results showed that MIND diet intervention significantly increased mean changes in the surface area of the inferior frontal gyrus (**A**) in comparison with the control group. The differences in cerebellum white matter (**B**) and cortex (**C**) in both groups are decreasing but not significant.

The whole-brain analysis did not indicate a significant effect for time × group interaction for cortical thickness, surface area, and cortical volume measures. It might be due to a few participants in each group and the short length of study. Also, we quantified the effect of diet intervention on Freesurfer generated cortical parcellation and subcortical segmentations as ROI analysis. Among cortical parcellation and subcortical segmentations, we selected areas of the brain (included: orbito-frontal cortex, inferior frontal gyrus, hippocampus, and cerebellum), which the effect of dietary patterns on them was examined previously^39,40^. To quantify the changes in brain structure between the two groups, we used the Man-Whitney u test. The result revealed significant changes in the surface area of the inferior frontal gyrus in the MIND diet group (Fig. 5A) as the surface area in this region increased in the MIND diet group and decreased in the control group (*p* = 0.018). It indicated that using a 3-month MIND diet can prevent surface area loss in the MIND group compared to the control group. Furthermore, the results showed a decrease in cerebellum white matter and cerebellum gray matter in two groups of studies (Fig. 5B, C). As can be seen in Fig. 5, the effect in the MIND group was more than the control groups.

## Discussion

In this 3-month randomized controlled study, we investigated the effect of the MIND diet on cognitive performances and brain volume changes in healthy obese women. To the best of our knowledge, this is the first study evaluating the effect of a short-term MIND diet intervention on cognition. Our results showed that the mean change of weight and percent of body fat were more decreased in the MIND diet group than the control group. Besides, group × time interaction revealed that in the MIND diet group, cognitive performance tests significantly improved relative to the control group. Our results also found that the 3 months intervention study can increase the volume of IFG in the MIND diet group between time points.

In order to comply with the ethical principles and adherence of the control group to stay in the study, we used a reduced-calorie diet in both groups, with the difference that in the control group, the hypo-calorie diet was accompanied by general recommendations. In the MIND group, the diet with reduced calorie intake comes with dietary recommendations of the MIND diet. Meals in both calorie-restricted diet groups included a balanced mix of foods with 50–55% carbohydrates, 30% fat, and 15–20% proteins. Therefore, in both groups, the average of reduced calories and macronutrient percentage have the same distribution.

A distinction can be considered between MIND diet-induced weight loss and weight reduced by calorie restriction. In addition, even a moderate weight loss of about 10% or less has been shown to lead to several health benefits that improve cognitive performance. In particular, our study showed that MIND hypocaloric diet intervention had been associated with more considerable improvement in weight and percentage of body fat than the hypocaloric diet alone. In line with this thought, the current previous randomized trial on 19 obese women showed that weight loss due to calorie restriction has a beneficial effect on brain structure^41^.

Thus, some of these changes may be due to weight loss. Still, better improvements in cognitive function following a MIND dietary pattern are due to specific dietary components in this pattern.

The results of our study indicate that adherence to the MIND diet has a more significant positive effect on FDST and BDST, which were used to measure verbal short memory. These findings are in line with a meta-analysis that revealed, adherence to the Mediterranean diet significantly improved working memory and global cognition compared to controls^42^. Amelioration in cognition performance was not specific for the MIND diet group alone and could be found in the control group too. Nonetheless, this improvement was significantly greater in the MIND diet group. In addition to enhancing working memory, we also received an improvement in Trail making test A that is considered executive function and verbal recognition memory concerning better performance on AVLT. Our findings are consistent with a community-based American cohort study that found a higher adherence to the Med diet was associated with a lower risk of developing MCI and slower rates of cognitive decline^43^. In contrast, an Australian cohort with an 8-year follow-up could not find an association between higher compliance of Med diet and cognition improvement^44^. Additionally^45^, demonstrated that the DASH diet, combined with aerobic exercise and reduced-calorie, was associated with advancing psychomotor speed performance than controls after the 4-month intervention. These inconsistent findings can be explained by the characteristics of the study populations, the long duration of the effect of nutritional changes on cognition, and the score used to evaluate adherence to the dietary patterns.

Regarding markers of neuronal factors, although no differences in mean plasma levels of BDNF were found after 3 months of intervention in volunteers allocated to the MIND diet group, analysis of the results showed higher overall plasma BDNF levels in the MIND diet group as compared with the control group. Our results are in line with the PREDIMED-NAVARA randomized trial, which showed that higher but not significant plasma BDNF levels were observed for participants assigned to both Mediterranean diet with nuts (Med + Nuts) and virgin olive oil (Med + VOO), specifically among patients with depression^46^. The role of the MIND diet on BDNF levels has been evaluated by lowering circulating levels of pro-inflammatory cytokines such as IL-6 and homocysteine, which could inhibit BDNF expression^46^. So, regarding this mechanism, adherence to a MIND diet is expected to be associated with higher plasma BDNF levels.

In addition, we also found a reduction in amyloid-beta and plasma levels of homocysteine in both intervention groups. Interestingly, a more significant decrease in homocysteine levels in the MIND diet group, indicating the impact of this intervention on improved cognitive performance when adhering to a hypocaloric diet. One of the most intriguing possible links between obesity and neurodegenerative diseases is that related to the attendance of high levels of homocysteine. Experimental studies reported homocysteine accumulated amyloid beta protein in the brain so that it may be involved in the destruction of DNA repair in hippocampal neurons, thus making them susceptible to the neurotoxicity effect of amyloid-beta protein^47^. These results are also consistent with a vast population study that demonstrated higher adherence to the Mediterranean diet significantly reduced levels of C-reactive protein, interleukin-6, and fibrinogen, as well as of homocysteine^48^. Recent publications from the PREDIMED study also found that nut consumption could inhibit the formation and defibrillation of amyloid-beta preformed fibrils^49^.

In the MIND diet group, our MRI data analysis showed an increase in gray matter volume in bilateral IFG. Our result was in line with Prehn's study, which shows the calorie restituted diet for 12-week improved recognition memory, paralleled by an increase in gray matter volume in IFG^41^. Similarly, a fMRI study of obese individuals declares that a 10% reduction in weight loss following a low-calorie diet for 6–8 weeks was accompanied by increased brain reward regions and decision-making systems such as the inferior frontal gyrus middle temporal gyrus^50^. Previous human studies declare that obesity can decrease the executive control of eating behaviors by reducing the inferior frontal gyrus activation^51^. Therefore, MIND diet intervention might enhance neuronal plasticity in frontal–temporal brain regions by increasing IFG volume. Besides, our study also resulted in a considerable reduction in gray and white matter volume of the cerebellum in both MIND and control groups. A review of 16 obese participants realized that they have higher relative brain white matter volume in the cerebellum and several brain regions, which can be partially decreased by a very low-calorie diet for 6 weeks^52^. fMRI studies investigate that the cerebellum is part of the bottom-up appetite control network, which might play a particular role in some cognitive performances, including interoceptive awareness, and has a role in determining energy needs and eating behavior^53^. Except for an increase in the gray matter volume of IFG, there was no group-by-time interaction in the rest of the regions in our study results. This is possible that the smaller sample size and short duration of our study missed statistical power to detect the effect of the MIND diet on brain structure volume.

Since both groups are received a calorie-restricted diet with the same distribution of macronutrients, the present study aimed to investigate whether the dietary components of the MIND pattern can improve cognitive function in obese individuals. The results of the present study showed a significant increase in the consumption of green leafy vegetables, other vegetables, berries, olive oil, fish, whole grains, and beans, as well as a significant decrease in butter, cheese, red meats, fast foods, and sweets in the MIND group compared with the control group.

Results revealed cognitive performances improved more in the MIND diet group in comparison with the control group. Participants in the MIND diet group consumed significantly more fiber and monounsaturated fatty acids while consuming less saturated fat and sugar. Although these changes were found in the control group, the variation in the MIND group was higher.

With regard to the mechanism, although the MIND diet was established on the component of the Mediterranean and DASH diets, it also has particular features that emphasize the consumption of berries, green leafy vegetables, and olive oils. As shown in previous studies, there was a linear relationship between the use of green leafy vegetables and slowing in cognitive decline. This study suggests that people who consume 1 to 2 servings of green leafy vegetables per day are 11 years younger than those who rarely or never consume^54^. Similar to our results, animal models' studies demonstrated that a higher intake of berries was associated with improved memory and learning. These beneficial cognitive effects of berries are also repeated in the Nurse Health Study^55^. Our findings are consistent with Washington heights-Inwood Community Aging Project results, showing that higher fish intake was positively associated with a larger mean cortical thickness^40^. Additionally, olive oil is one of the essential key elements of the MIND diet pattern. In line with our results, a randomized trial in the sub-study of PREDIMED showed that Mediterranean intervention supplemented with extra virgin olive oil was impressive in higher cognitive scores than a low-fat diet among Spaniards at high cardiovascular risk^56^.

On the other hand, restrictions on the intake of red meat, saturated fats, and pastries are other essential components of the MIND pattern. These components can have detrimental effects on the cardiovascular system and consequently have been related to a more considerable cognitive decline and risk of dementia^57^. As a result, these nutrients may have an independent action mechanism that synergistically protects against neurological pathogenesis. Also, it can be speculated that these components of the MIND diet could be found to protect the brain with their antioxidant and anti-inflammatory properties to protect against obesity.

The strengths of this trial included: To our knowledge, this study was the first RCT to consider the effect of MIND diet intervention on cognitive performance and brain structure among healthy obese adults. Additionally, the present study was an entirely controlled intervention study. A well-informed nutrition consultant attentively considers all efforts to adhere to the dietary patterns at regular intervals. Finally, we used a comprehensive cognitive test battery delineated to correlate with dietary patterns in previous systematic reviews in the current study.

Our study also has limitations that must be considered when interpreting data. First, it should be noted that the short study length, along with the relatively small sample size, may not allow us for further comparisons with adequate statistical power to establish between specific subgroups. However, it is consistent with sample sizes in further studies examining the effect of different dietary patterns on cognitive performance. Second, the study sample only examined a particular population and may not display the broader adult population with varying levels of education and health. However, this relative homogeneity of study participants can be attributed to the strength of the current study.

## Conclusion

In conclusion, the results of this randomized controlled study have sought to consolidate the hypothesis that shows for the first time in humans a beneficial effect of MIND diet on cognition and brain structure in obese adults. In particular, the results demonstrated that these effects were specific for minimal to marked weight loss, which may have a highlighted impact on dietary patterns and cognitive performance simultaneously. According to the current development of obesity in the present century and its threatening effect on the neuronal system in adults, the strategies that focus on the reduction of stress reactivity and modulate structural functions should be viewed as more effective than pharmacological approaches. In consequence, exploring the validity of our findings in larger study samples as well as longer durations will assist researchers in developing a clear understanding of whether or not a MIND diet intervention has an evidence-informed effect on cognitive function.
